# Extracellular Vesicle Mitochondrial DNA Reflects Podocyte Mitochondrial Stress and Is Associated with Relapse in Nephrotic Syndrome

**DOI:** 10.3390/ijms26157245

**Published:** 2025-07-26

**Authors:** Robert L. Myette, Chet E. Holterman, Mayra Trentin-Sonoda, Tyler T. Cooper, Gilles A. Lajoie, George Cairns, Yan Burelle, Nour El Khatib, Joanna Raman-Nair, Dylan Burger, Christopher R. J. Kennedy

**Affiliations:** 1 Division of Pediatric Nephrology, Children’s Hospital of Eastern Ontario Research Institute & Children’s Hospital of Eastern Ontario, University of Ottawa, Ottawa, ON K1H 8L1, Canada; rmyette@cheo.on.ca; 2 Kidney Research Centre, Ottawa Hospital Research Institute, University of Ottawa, Ottawa, ON K1N 6N5, Canada; cholterm@uottawa.ca (C.E.H.); MTrentinSonoda@cheo.on.ca (M.T.-S.); nelkh031@uottawa.ca (N.E.K.); jramann2@uottawa.ca (J.R.-N.); ckennedy@uottawa.ca (C.R.J.K.); 3 Department of Cellular & Molecular Medicine, Faculty of Medicine, University of Ottawa, Ottawa, ON K1N 6N5, Canada; 4 Department of Biomedical and Molecular Sciences, Queen′s University, Kingston, ON K7L 3N6, Canada; tcoope2@uwo.ca; 5 Department of Biochemistry, The University of Western Ontario, London, ON N6A 3K7, Canada; glajoie@uwo.ca; 6 Interdisciplinary School of Health Sciences, Faculty of Health Sciences, University of Ottawa, Ottawa, ON K1N 6N5, Canada; gcairns@uottawa.ca (G.C.); yburell2@uottawa.ca (Y.B.); 7 Department of Biochemistry, Microbiology and Immunology, University of Ottawa, Ottawa, ON K1N 6N5, Canada; 8 Center for Neuromuscular Disease (CNMD), Faculty of Medicine, University of Ottawa, Ottawa, ON K1H 8M5, Canada

**Keywords:** nephrotic syndrome, pediatrics, extracellular vesicles, mitochondria

## Abstract

Idiopathic childhood nephrotic syndrome is a common glomerulopathy comprising proteinuria, hypoalbuminemia, and edema. Podocyte dysfunction is central to this disease process. Extracellular vesicles are released from stressed cells and can represent a molecular snapshot of the parent cell of origin. We previously showed that urinary large extracellular vesicles (LEVs) derived from podocytes are increased in patients with nephrotic syndrome relapse. Here, we investigated the role of mitochondrial DNA (mtDNA) within LEVs both in vitro and in vivo, revealing the novel finding that podocytes release LEVs containing mtDNA, driven by mitochondrial stress. A puromycin aminonucleoside nephrosis rat model showed foot process effacement on electron microscopy and urinary LEVs with significantly increased mtDNA. Prednisolone, which drives remission in nephrotic syndrome in children, attenuated mitochondrial stress and reduced the amount of mtDNA content within LEVs in vitro. Lastly, urinary LEVs from children with nephrotic syndrome also contain mtDNA, and it is the podocyte LEV-fraction which is preferentially enriched. Overall, these data support a potential mechanism of podocyte mitochondrial stress in non-genetic, idiopathic pediatric nephrotic syndrome.

## 1. Introduction

Idiopathic nephrotic syndrome (iNS) is a prevalent glomerulopathy in children and comprises proteinuria, hypoalbuminemia, and edema [1], with the most common form having minimal change lesions on histopathology. Several etiologies of iNS have been proposed, and many implicate different components of the immune system; however, our understanding of downstream signaling within the podocyte, in the context of iNS and other glomerulopathies, is still significantly lacking.

Podocytes are terminally differentiated epithelial cells and are indispensable as key members of the glomerular slit diaphragm [2,3]. Indeed, podocytes act not only as support structures for appropriate filtration; they have also been shown as robust communicators with the underlying glomerular slit diaphragm and are capable of antigen presentation and signaling via common immune pathways [4,5,6,7,8].

Mitochondria are responsible for energy production in the form of ATP, and they maintain their own circular DNA [9]. They also act as signaling hubs, contributing to important immune crosstalk within the cell [10]. The podocyte has fewer mitochondria than tubular cells, for example, which has prompted some groups to hypothesize that podocytes are less reliant upon mitochondria for energy needs [11]. A study by Brinkkoetter et al. [11] revealed that the perturbation of mitochondrial biogenesis, inducing defective fission-fusion capacity, and reduced stability in mitochondrial DNA (mtDNA) did not lead to a pathologic phenotype under physiological and non-stressed conditions. Contrarily, other groups showed that changes to mitochondrial aerobic respiration does lead to a pathologic phenotype seen in diseases such as diabetic kidney disease and focal segmental glomerulosclerosis [12]. It is therefore tempting to hypothesize that podocytes rely on anaerobic respiration during times of stability, switch to a mode requiring more energy, and thus become more dependent on mitochondria during times of stress. With regard to their roles in immune signaling, mitochondria can respond to exogenous stimuli, and when damaged, can secrete damage-associated molecular patterns (DAMPs), including mtDNA, which interact with pattern recognition receptors (PRRs) [13]. These PRRs include the cyclic GMP-AMP synthase–stimulator of interferon genes (cGAS-STING) and Toll-like Receptor 9 (TLR9) [13], all of which are found within podocytes.

Extracellular vesicles (EVs) are small, membrane-enclosed effector molecules released from cells. Some EVs are actively shed from their parent cell of origin, are ~100–1000 nm in size, and are preferentially isolated following a 20,000× *g* centrifugation (called large extracellular vesicles (LEVs) here-forward) [14]. LEVs retain characteristics of the parent cells and are packaged with biomolecules including DNA. LEVs are released from stressed cells and can act as liquid biopsies. Indeed, we showed recently that children with iNS have elevated levels of podocyte LEVs in their urine during relapse, and these podocyte LEVs are reduced to near-zero levels during remission [15].

The role of mitochondria, and particularly of mtDNA in the pathogenesis of MCD is an underexplored area of research. The content of EVs has been studied extensively, and lately, there has been a particular interest in mtDNA within EVs (including plasma EVs and monocytic EVs) reflecting mitochondrial stress [16,17,18]. We therefore sought to determine if mtDNA was present in LEVs from human podocytes (hPods) in vitro as a marker of mitochondrial stress. We used puromycin aminonucleoside (PAN) as a podocyte toxin and accepted cellular model of nephrosis as it also induces mitochondrial stress [19,20,21,22,23].

We discovered that the mitochondrial oxygen consumption rate is increased in hPods in the presence of PAN, and this is associated with a marked increase in mtDNA within LEVs from hPods, hinting at a possible unique mechanism of podocyte injury in iNS. We next observed mtDNA within urinary LEVs from rats following treatment with PAN, associating with podocyte foot process effacement on electron microscopy (EM) and significant proteinuria. Lastly, in a cohort of children with iNS we observed elevated levels of mtDNA in urinary LEVs, and preferentially those from podocytes, during relapse. We show here for the first time the novel use of digital droplet PCR (ddPCR) in the quantification of mtDNA in urinary LEVs from children with iNS, possibly representing a novel approach to disease monitoring.

## 2. Results

**Figure 1:**
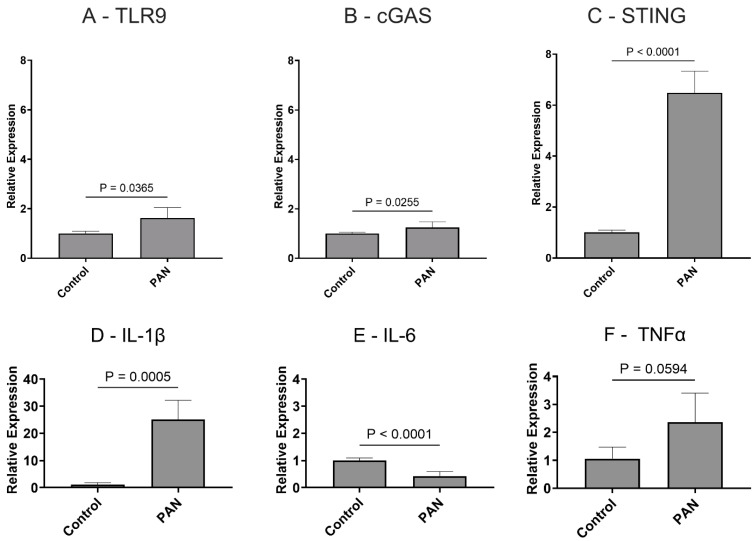
**Puromycin aminonucleoside (PAN) significantly induces specific pattern recognition receptor genes, as well as some of their downstream effectors in conditionally immortalized human podocytes (hPods)**. (**A**) Toll like receptor 9 (TLR9) was significantly induced following PAN treatment (25 μg/mL; *n* = 3) compared to control (*n* = 3). *p* = 0.0365, two tailed *t* test. (**B**) cGAS, cyclic GMP-AMP synthase, was similarly induced following PAN treatment (25 μg/mL; *n* = 3) compared to control (*n* = 3). *p* = 0.0255, two tailed *t* test. (**C**) STING, stimulator of interferon genes, was most prominently induced following PAN (25 μg/mL; *n* = 3) compared to control (*n* = 3). *p* < 0.0001, two tailed *t* test. Next, we assessed downstream effectors, including (**D**) IL-1ß, Interleukin 1-beta, which was significantly induced following PAN treatment (25 μg/mL; *n* = 3) compared to control (*n* = 3). *p* = 0.0005, two tailed *t* test. IL-6, interleukin 6 (**E**), was significantly decreased following treatment with PAN (25 μg/mL; *n* = 3) compared to control (*n* = 3). *p* < 0.0001, two tailed *t* test. TNF-alpha levels were unaffected by PAN treatment (**F**). Relative expression = relative fold change using the delta-delta CT method with glyceraldehyde 3-phosphate dehydrogenase (GAPDH) as a housekeeping gene.

**PAN induces global oxidative stress in hPods which impacts PRR gene expression levels:** Following PAN treatment, mean malondialdehyde (MDA) levels were elevated when compared to control hPods. The mean MDA level for control hPods was 0.17 μM compared to 0.29 μM following PAN treatment (*p* = 0.01). This indicated that PAN treatment induced oxidative stress in podocytes in vitro. Next, we assessed the impact of PAN treatment on PRR gene expression, including TLR9 and cGAS-STING. PAN increased TLR9 (*p* < 0.05; Figure 1A), cGAS (*p* < 0.05; Figure 1B), and STING (*p* < 0.01; Figure 1C) mRNA levels, relative to control.

IL-1ß mRNA levels are increased, but IL-6 levels are decreased, and TNF-alpha levels are minimally increased following PAN treatment: We next looked at downstream inflammatory signatures associated with PRR signaling and discovered that 24 h of PAN treatment leads to an upregulation of IL-1ß mRNA compared to control (*p* < 0.0001; Figure 1D). IL-6 decreased significantly (*p* < 0.0001; Figure 1E) and TNF-alpha mRNA levels were borderline increased (*p* = 0.059, Figure 1F).

**Figure 2:**
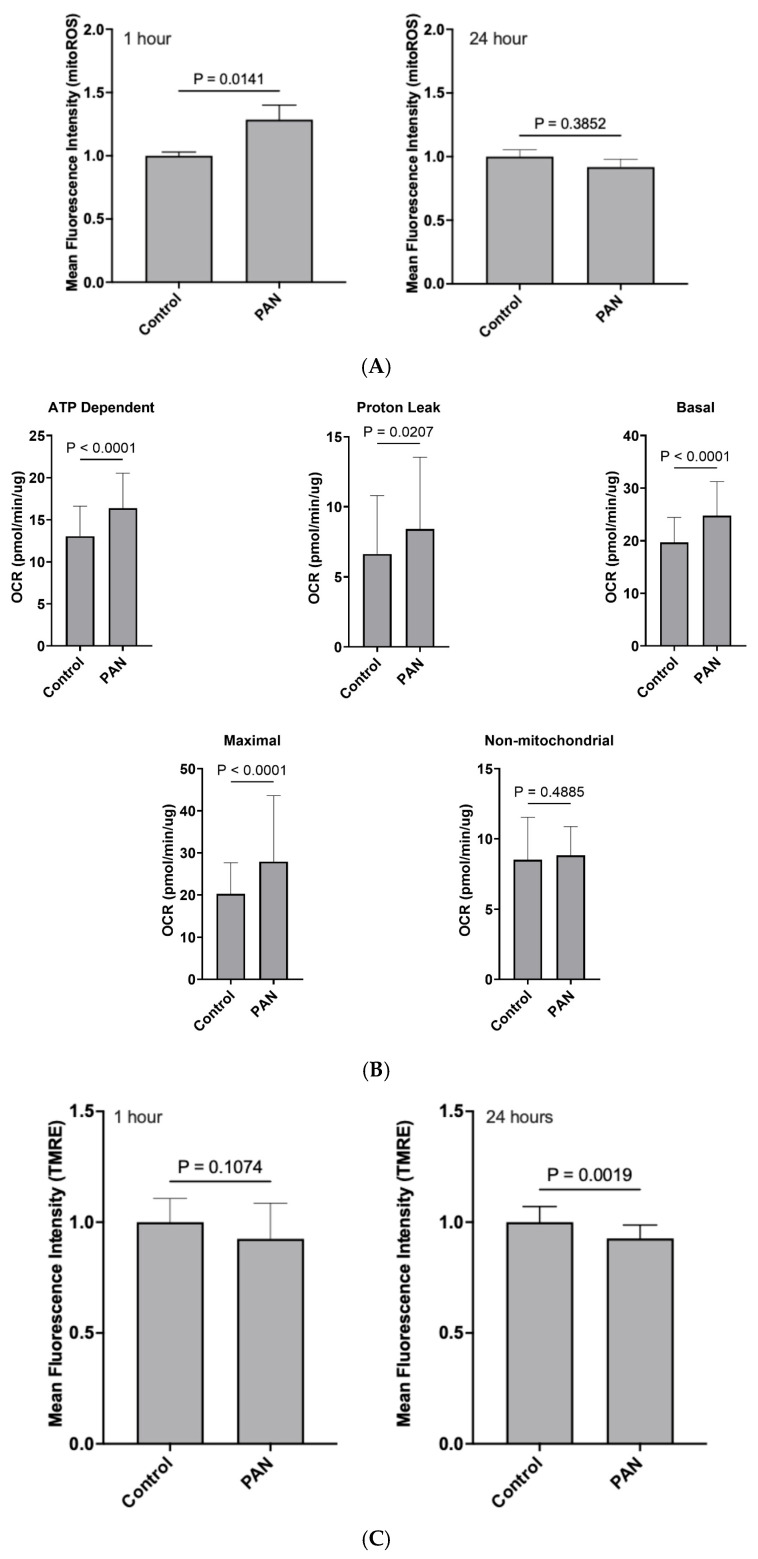

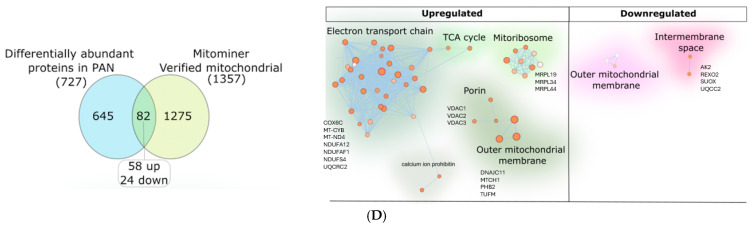
**Mitochondrial dynamics are altered following treatment of conditional immortalized human podocytes with PAN** (**A**) Mitochondrial ROS (mitoROS) production following PAN treatment of hPods for 1 h, or 24 h. mitoROS was significantly increased following 1 h of PAN treatment (25 μg/mL, *n* = 6) compared to control (*n* = 6). *p* = 0.0141, two tailed *t* test. (**B**) The impact of 1 h puromycin aminonucleoside (PAN, 25 μg/mL) treatment on oxygen consumption rate (OCR) using the Seahorse Assay. Basal oxygen consumption rate was increased following treatment with PAN (25 μg/mL, *n* = 3, *p* < 0.0001, two tailed *t* test). Similarly, for ATP Dependent OCR, we observed an increase following PAN (25 μg/mL, *n* = 3, *p* < 0.0001, two tailed *t* test). Maximal OCR was increased following treatment with PAN (25 μg/mL, *n* = 3, *p* < 0.0001, two tailed *t* test). There was no impact on non-mitochondrial OCR following treatment with PAN (25 μg/mL, *n* = 3). There was an increase in Proton Leak (*p* = 0.02). (**C**) Tetramethyl rhodamine, ester (TMRE) assay revealing decreases in mitochondrial membrane potential (MMP) following 24 h of PAN treatment, but not 1 h. Specifically, after 24 h of PAN (25 μg/mL) there was a significant decrease in mean fluorescence intensity representing decreased MMP (*n* = 6, *p* = 0.0053, two tailed *t* test) (**D**) Proteomic assessment of hPods following treatment with PAN (25 μg/mL, *n* = 4), compared to control (*n* = 4). 727 proteins were differentially abundant, with 82 overlapping with the MitoMiner database (https://www.mrc-mbu.cam.ac.uk/research-resources-and-facilities/mitominer, accessed on 28 January 2025). Of the 82 overlapping proteins, 58 were upregulated, and 24 were downregulated. Upregulated proteins comprised those associated with the electron transport chain (cytochrome c oxidase subunit 6C, COX6C; mitochondrially encoded cytochrome b, MT-CYB; mitochondrially encoded NADH dehydrogenase 4, MT-ND4; NADH dehydrogenase [ubiquinone] 1 alpha subcomplex subunit 12, NDUFA12; NDUFAF1, NDUFS4, Ubiquinol-Cytochrome c Reductase Core Protein 2, UQCRC2), mitoribosomes (Mitochondrial Ribosomal Protein L19, MRPL19; MRPL34, MRPL44), porins (Voltage-Dependent Anion Channel 1, VDAC1; VDAC2, VDAC3), and the outer mitochondrial membrane (DnaJ Heat Shock Protein Family (Hsp40) Member C11, DNJAC11; Mitochondrial Carrier Homolog 1, MTCH1; Prohibitin 2, PHB2; Tu Translation Elongation Factor, Mitochondrial, TUFM), for example. Those associated with downregulation were related to the outer mitochondrial membrane, and intermembrane space (Adenylate Kinase 2, AK2; RNA Exonuclease 2, REXO2; Sulfite Oxidase, SUOX; Ubiquinol-Cytochrome c Reductase Complex Assembly Factor 2, UQCC2), for example. (To be paneled). (**A**)Mitochondrial ROS production following treatment of hPods with PAN, relative to control. (**B**) Seahorse assay comparing control to PAN treated hPods. (**C**) TMRE assay comparing control hPod cells with PAN treated cells. (**D**) Proteomic assessment of hPods following treatment with PAN, compared to control.

**PAN treatment led to an increase in mitochondrial-specific reactive oxygen species (mitoROS) at 1 h, but not 24 h:** We observed that following a 1 h PAN treatment, there was an obvious spike in mitoROS production when compared to control (*p* < 0.05; Figure 2A); however, this was attenuated by 24 h (*p* > 0.05; Figure 2A).

**PAN induced an acute bioenergetic stress that subsides over time:** Oxygen consumption was then monitored to assess the impact of PAN on mitochondrial bioenergetics. Acute 1 h exposure of hPods to PAN induced a rapid increase in baseline respiration indicating that PAN’s impacts on mitochondria are immediate. This was caused by stimulation of oxidative phosphorylation (OXPHOS), as evidence by an increased oligomycin-sensitive respiration. Furthermore, PAN also induced a significant increase in OXPHOS-independent mitochondrial respiration, pointing to an increased proton leak through the inner membrane. Maximal respiration induced by uncoupling with the protonophore Carbonyl cyanide-p-trifluoromethoxyphenylhydrazone (CCCP) was also increased following 1 h treatment with PAN (Figure 2B). In contrast, aside from a mildly increased proton leak, no discernable differences in mitochondrial respiratory parameters were observed following 24 h exposure to PAN. Bioenergetics were also assessed by monitoring mitochondrial membrane potential (MMP) with Tetramethyl rhodamine ethyl ester (TMRE). As shown in Figure 2C, MMP followed a downward trend after 1 h exposure to PAN and became significantly lower after 24 h. Altogether, these results suggest that PAN induced a rapid and robust bioenergetic stress that subsided over time.

**Protein levels of Complexes I-V of the electron transport chain and total mitochondrial mass, fission, and fusion machinery are seemingly unaffected by PAN treatment:** We next sought to evaluate differences in oxidative phosphorylation proteins using a total OXPHOS Western blot antibody cocktail. We did not observe differences in protein expression for each complex between control and 24 h PAN treatments. We next assessed the impact of 24 h PAN treatments on TOM20 (Translocase of the outer mitochondrial membrane 20; total mitochondrial mass), DRP1 (Dynamin-related protein 1; fission), and MFN1 (Mitofusin 1; fusion). We initially observed a decrease in MFN1 protein expression relative to control; however, this observation was attenuated when normalizing for total mitochondrial mass (MFN1/TOM20).

**hPod proteomics revealed differences in other mitochondrial proteins following 24 h of PAN treatment:** We next applied gas-phase fractionation combined with data-independent acquisition (GPF-DIA) proteomics to the hPods treated with PAN to specifically determine changes to mitochondrial proteins in a more sensitive manner. Specifically, we identified 4531 total proteins, of which, 727 were differentially abundant proteins following PAN treatment. Next, utilizing MitoMiner (https://www.mrc-mbu.cam.ac.uk/research-resources-and-facilities/mitominer, accessed on 28 January 2025), a mitochondrial protein database, we determined that 82 differentially abundant proteins were mitochondrial in nature, and of those, 58 were upregulated, and 24 were downregulated (Figure 2D). Many of the proteins were associated with the electron transport chain, the mitochondrial membrane, mitochondrial inner membrane, and the mitochondrial matrix, among others (Figure S1A,B). Specifically, we observed significant upregulation of 58 proteins, including voltage dependent anion channel (VDACs) 1 (*p* < 0.05), 2 (*p* < 0.001), and 3 (*p* < 0.05; Figure 2D) and several mitochondrial ribosomal proteins (MRPLs), including MRPL19 (*p* < 0.05), MRPL34 (*p* < 0.05), and MRPL44 (*p* < 0.05). TFAM was also significantly upregulated (*p* = 0.02) following PAN treatment. We also observed 24 significantly downregulated mitochondrial proteins. Altogether, this suggests that PAN-injury leads to mitochondrial stress.

**Figure 3:**
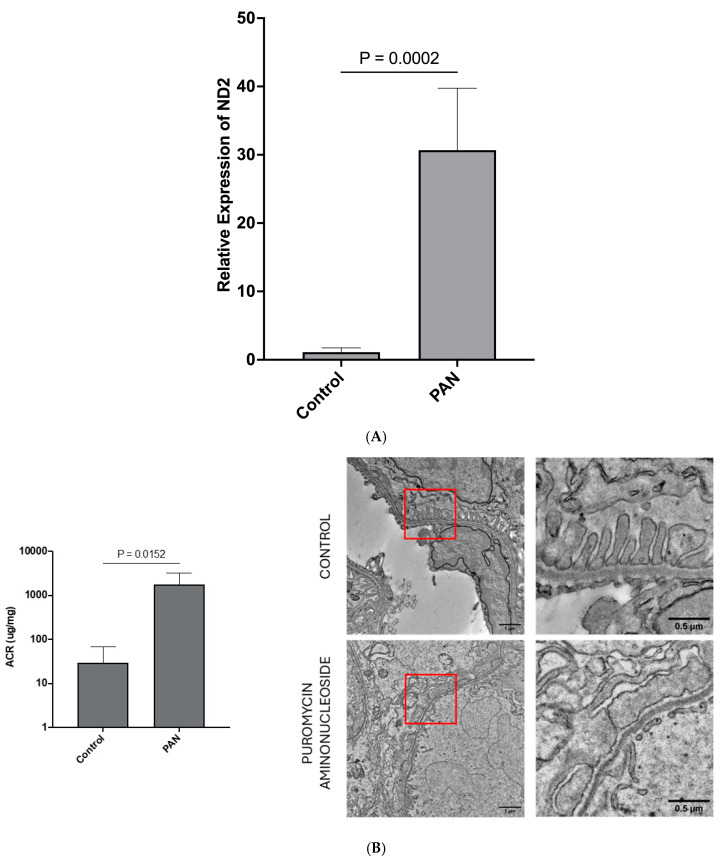

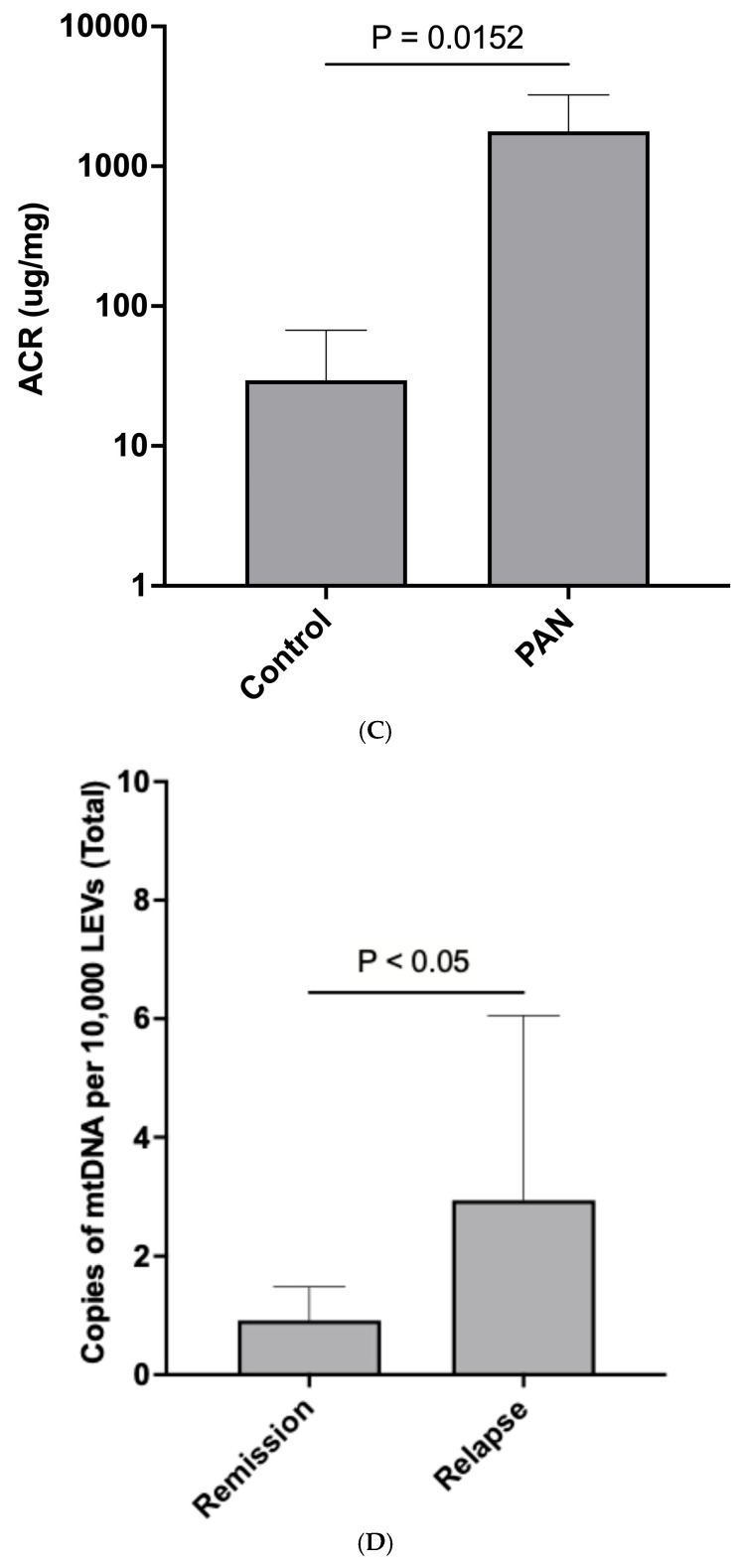
**Large extracellular vesicles (LEVs) from conditionally immortalized human podocytes contain significantly elevated mtDNA content after treatment with PAN, and this is recapitulated in a rat nephrosis model.** Further, urinary LEVs from children with nephrotic syndrome also have elevated levels of mtDNA (**A**) Podocyte LEV mtDNA content is markedly elevated after 24 h of treatment with PAN (25 μg/mL, *n* = 6) compared to control (*n* = 3). *p* = 0.0002, two tailed *t* test. (**B**) Sprague-Dawley rats exposed to PAN (100 mg/kg, single injection day 0; *n* = 6) were significantly more albuminuric at Day 9 when compared to control animals (*n* = 6). *p* = 0.0152, two tailed *t* test. Podocyte foot processes were preserved on electron microscopy for control animals (*n* = 6); however, for PAN-treated animals (*n* = 6), the foot processes were completely effaced. Representative images presented here, red boxes are magnified in images to the right. This observation was present in all EM images obtained. (**C**) Urinary LEVs from rats were markedly enriched with mtDNA following treatment with PAN (Day 9 shown; *n* = 6 PAN, *n* = 6 control). *p* = 0.0095, two tailed *t* test (**D**) mtDNA in total urinary large extracellular vesicles obtained from children with iNS in both stages of relapse (*n* = 11) and remission (*n* = 11). The results presented are copies of mtDNA per 10,000 total LEVs. *p* < 0.05, two tailed *t* test. (To be paneled). (**A**) Podocyte LEV mtDNA content is markedly elevated after 24 h of treatment with PAN. (**B**) Sprague-Dawley rats exposed to PAN were significantly more albuminuric at Day 9 when compared to control animals. (**C**) Urinary LEVs from rats were markedly enriched with mtDNA following treatment with PAN. (**D**) mtDNA in total urinary large extracellular vesicles obtained from children with iNS in both stages of relapse and remission

**LEVs from stressed hPods are enriched in mtDNA following treatment with PAN:** Having established that mitochondrial stress is present in hPods treated with PAN, we next hypothesized that mtDNA would be present in hPod-LEVs and would associate with hPod injury. We therefore assessed the presence of two mitochondrial genes within LEVs from hPods (NADH Dehydrogenase Subunit 2 (ND2), and Cytochrome C Oxidase 2 (COX2)). Following preliminary studies showing a similar trend in both genes we focused on a single gene, ND2. The LEV-ND2 gene content was higher following 24 h PAN treatment compared to control (*p* = 0.0002; Figure 3A). We next explored whether LEVs from the urine of rats treated with PAN contained mtDNA.

**Sprague-Dawley (SD) rats are susceptible to PAN, are grossly albuminuric after PAN treatment, and have significant podocyte foot process effacement on EM:** Following treatment with PAN, there were no obvious differences between the control animals and those treated with PAN at Day 0. However, at the time of euthanasia and organ harvesting (Day 9), it was apparent that those animals who received PAN had ascites. At endpoint (Day 9), urinary ACR was elevated in those animals who received PAN compared to control animals (*p* < 0.05; Figure 3B). Representative transmission EM images revealed classic podocyte ultrastructure in control animals (Figure 3B), while those treated with PAN had significant podocyte foot process effacement (Figure 3B).

**Urinary LEVs from SD rats treated with PAN have increased levels of mtDNA:** We next isolated urinary LEVs from both PAN-treated and control rats. Relative to control animals, at day 9, PAN-treated rat urinary LEV mtDNA levels were ~1000 fold higher (*p* = 0.0087, Figure 3C). Importantly, there was not enough urine to do podocyte-specific LEV pulldowns to determine the relative amounts of mtDNA in total LEVs versus podocyte-specific LEVs. Given the above observations, we next returned to our patient cohort and examined mtDNA levels in urinary LEVs from children with iNS.

**Mitochondrial DNA is enriched in LEVs from patients with MCD:** We compared the biochemical parameters between 11 patients when they were in relapse vs. remission, and no significant differences were observed in renal function or blood pressure between these states [15]. LEVs were isolated from urine samples provided by children with nephrotic syndrome enrolled in this prospective study. We previously showed that urinary, podocyte-specific LEVs were increased in the urine of children with nephrotic syndrome relapse. Using both qPCR and ddPCR, we show here that the mtDNA is present in total urinary LEVs and associates with disease relapse in MCD. The mean copy number per 10,000 total LEVs was 2.9 (±3.1) in relapse and 0.92 (±0.6) in remission (*p* < 0.05; Figure 3D).

**mtDNA is markedly increased in urinary podocyte LEVs compared to all other urinary LEVs:** We next pooled relapse urine samples, and separately, remission urine samples. LEVs were isolated and treated with DNase and then subjected to magnetic bead conjugation with Anti-Podoplanin antibody to select for podocyte-specific LEVs. We then isolated the DNA from the LEVs and performed ddPCR to quantify mtDNA copy number. We observed that total urinary LEVs increased approximately 33-fold in relapse, and this was coupled to a 4-fold increase in mtDNA copies per LEV. Podocyte-specific LEVs increased approximately 9-fold, with a 3-fold increase in mtDNA copy number per LEV. Relatively, we observed more mtDNA species per LEV from podocytes.

**Figure 4:**
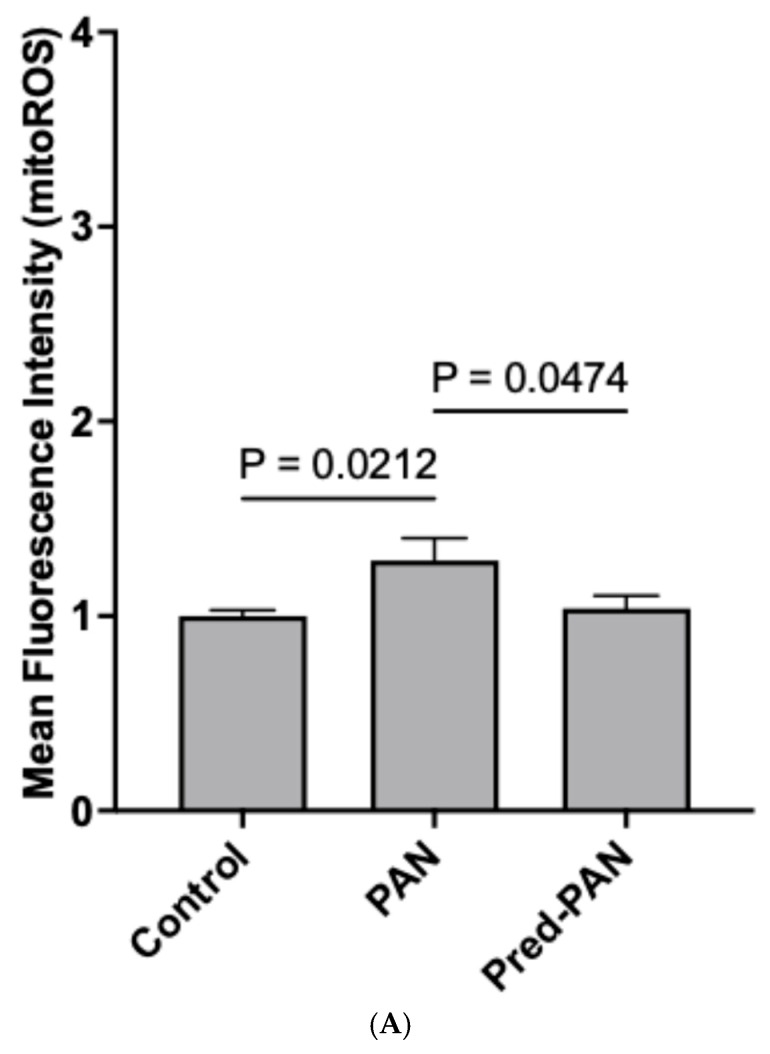

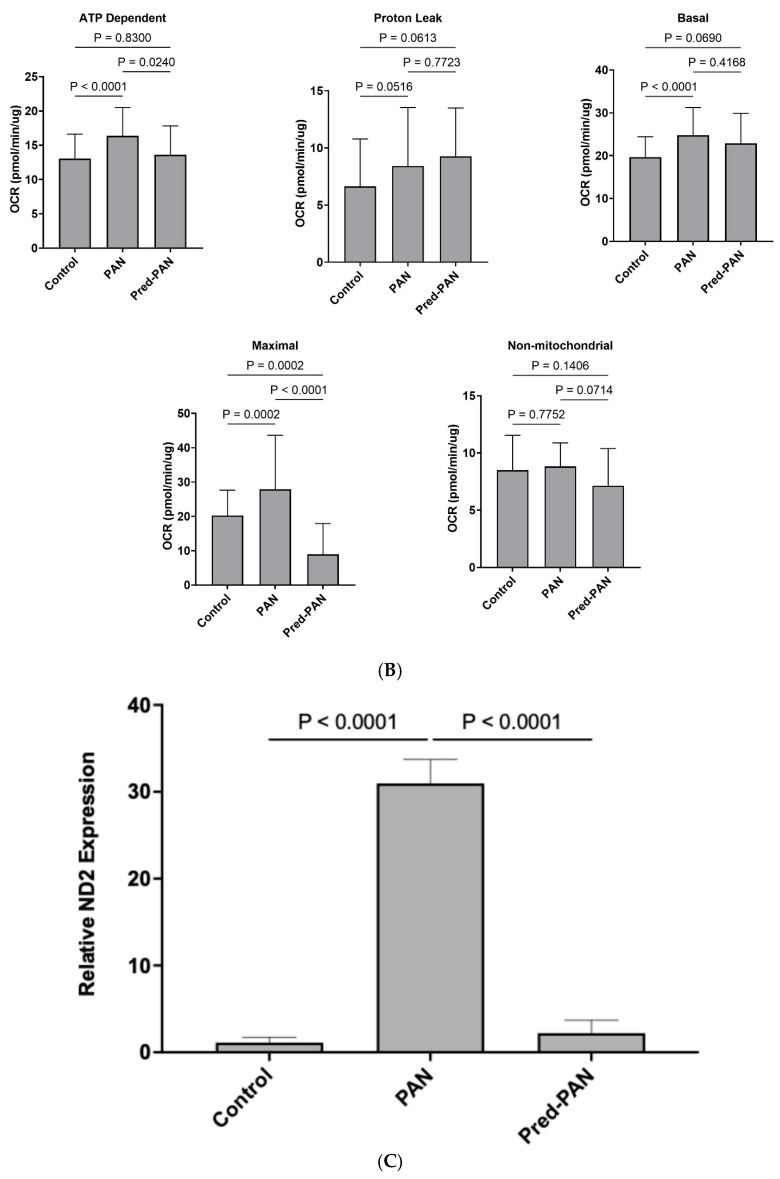
**Impact of prednisolone pre-treatment on mitoROS production, increases in OCR, and mtDNA within LEVs.** (**A**) Pre-treatment with prednisolone (1 μM) prevented the increases in mitoROS observed following treatment with PAN (1 h, 25 μg/mL, *n* = 6). *p* = 0.0474, one way ANOVA. (**B**) Similarly, increases in ATP dependent and maximal OCR following PAN treatment (1 h, 25 μg/mL, *n* = 3) were attenuated with pre-treatment with prednisolone (1 μM, *n* = 3). *p* < 0.0001, one way ANOVA (**C**) Lastly, a significant reduction in the amount of mtDNA species in LEVs from hPods pre-treated with prednisolone (1 μM; *n* = 6) prior to PAN (25 μg/mL, *n* = 6), relative to control (*n* = 3). *p* < 0.0001, one way ANOVA. (To be paneled).(**A**) Impact of prednisolone pre-treatment on mitoROS production in hPods. (**B**) Prednisolone pre-treatment attenuates increases in OCR in hPods following PAN treatment. (**C**) There is a significant reduction in the amount of mtDNA species in LEVs from hPods pre-treated with prednisolone prior to PAN injury.

**Prednisolone decreases oxidative stress, hPod LEV-mtDNA content, and mitochondrial stress in vitro:** Lastly, given its role as the gold standard therapy for children with iNS, and the presence of glucocorticoid receptors on podocytes, we assessed the effectiveness of prednisolone treatment in our cell culture model. When reanalyzing our data from Figure 2 and Figure 3, we observed that pre-treatment with prednisolone inhibited the production of mitoROS following a 1 h treatment with PAN (*p* < 0.05; Figure 4A). Further, pre-treatment of hPods with prednisolone attenuated the change in some but not all mitochondrial bioenergetic parameters observed in response to PAN (Figure 4B). Specifically, prednisolone treatment dampened the rise in ATP=dependent and maximal OCR observed in response to PAN treatment without significantly affecting proton leak, basal and non-mitochondrial OCR (Figure 4B). Lastly, prednisolone pre-treatment attenuated the packaging of mtDNA into LEVs (Figure 4C).

## 3. Discussion

The key findings from this study include the novel discovery of mtDNA within LEVs from stressed podocytes in vitro, a recapitulation of these findings in a rat nephrosis model, and lastly, the observation that urinary LEVs from children with iNS and relapse have increased mtDNA compared to remission. Using podocyte-specific magnetic beads, we showed that there was enrichment of mtDNA in podocyte EVs compared with other urinary EV populations in children with iNS. We previously showed that podocyte-specific LEVs are increased in the urine from children with iNS during times of relapse, and that these LEV levels were drastically reduced in remission [15]. The presence of mtDNA within non-podocyte EVs has been described by our group [24] and others [25,26]; however, this is the only description of LEV-mtDNA in podocytes to our knowledge.

Herein, we have further linked the early production of mitoROS and early increases in OCR followed by later findings of mitochondrial membrane depolarization in hPods treated with PAN. This suggests that PAN may induce mitochondrial stress at an early time point leading to an initial overactive phenotype, perhaps related to compensation as a result of cellular stress [27]. Indeed, after 1 h of PAN treatment, we observed increases in ATP dependent, basal, and maximal OCR when compared to control cells. The timing aligns with an early increase in mitoROS, which may be a result of the increase in mitochondrial OCR. Interestingly, there is an increase in proton leak after 1 h of PAN, relative to control, which may be an attempt to buffer ROS production [28], and the persistence of a mild proton leak at 24 h ties together with the lower MMP at 24 h and might represent a mild yet persistent signature of cell stress and bioenergetic dysfunction.

In association with these changes, we have observed that PRR genes are induced, suggesting an increase in mtDNA within the cytosol. Within the podocyte, there is ample evidence that cytosolic mtDNA elicits immune responses via inherent immune pathways, including cGAS-STING and TLR9 [6,29,30,31,32]. Herein, we describe increases in TLR9 and cGAS-STING mRNA levels following treatment with PAN. We further confirmed that PAN induces oxidative stress in hPods which associates with the induction of TLR9, and cGAS-STING mRNA expression. Ultimately, expression of the downstream effector IL-1β is induced. This is consistent with prior reports by Mitrofanova et al., who showed that podocytes express all components of the cGAS-STING signaling pathway [30].

Our results suggest that PAN-induced podocyte injury leads to extrusion of mtDNA into the cytosol with subsequent packaging into LEVs. The process by which mtDNA is released from mitochondria is not completely elucidated; however, it is thought that this can occur either via transport pores in the outer mitochondrial membrane [33], or, via fission events that cause mtDNA to leak from the mitochondria into the cytosol [34]. We posit that in the context of PAN-induced podocyte injury this occurs via mitochondrial membrane pores as we did not see any changes in fission and fusion machinery. It is therefore possible that our novel finding of mtDNA within podocyte LEVs may represent a unique self-preservation mechanism. Importantly, our work shows that preventative treatment with prednisolone led to a decrease in mitoROS and attenuated increased OCR in the context of CCCP treatment. This is perhaps not surprising given that podocytes express the glucocorticoid receptor [35]. In turn, prednisolone treatment of podocytes prior to injury with PAN led to a marked reduction in the amount of LEV-mtDNA. Consistent with this, we saw similar reductions in LEV-mtDNA from the urine of children with nephrotic syndrome who entered remission following treatment with prednisone.

We next sought to determine if LEVs are packaged with mtDNA in a rodent model of nephrotic syndrome, the PAN-nephrosis model [36,37]. Indeed, the SD rats in our cohort were significantly nephrotic, with ascites, and elevated urinary ACR. Coupled with this significant proteinuria was an ~1000-fold increase in LEV-mtDNA species when compared to control animals injected with saline. To our knowledge, this is the first time that LEVs have been isolated from rat urine and their mtDNA content examined. While the direct mechanism of mtDNA packaging remains elusive, this appears to be a conserved mechanism, observed in both human podocytes and this murine model, as well as in children with iNS.

Our paper is not without limitations. Much of this work was performed using conditionally immortalized hPods which may not accurately reflect the glomerular environment. Further, we were unable to determine whether the mtDNA-containing urinary LEVs in our rat model were of podocyte origin. Conversely, the strengths of this paper include the use of in vitro and in vivo studies, as well as human specimens to describe a conserved mechanism associated with podocyte stress in iNS. Much work remains to understand how the mtDNA is extruded from hPod mitochondria. Our proteomic data hints at a potential mechanism, specifically, VDAC1. We observed increases in VDAC1 levels in the cellular proteome, indicating an increase in protein expression following PAN treatment. Given reports that VDAC1 can interact with mtDNA, and VDAC oligomers have been shown to form mitochondrial pores to release mtDNA fragments, we speculate this may represent the mechanism by which mtDNA is extruded from mitochondria into the cytosol in living cells [33]. By showing that LEV-mtDNA levels are increased in podocytes in vitro, in the urine of rats with nephrotic syndrome, and then in children with iNS, we have provided a foundation for future mechanistic studies.

In summary, the key findings from this study include the novel discovery of mtDNA within LEVs from stressed podocytes in vitro, in a rat nephrosis model, and in children with iNS. Our data suggests that early mitochondrial stress drives an increase in OCR in this model, with subsequent mitochondrial membrane depolarization. We posit that this results in mtDNA extrusion which triggers packaging into LEVs for release. Prednisolone appears to prevent this process from happening; however, it remains unclear whether it is possible to more specifically target the mtDNA release. Likewise, whether this is specific to iNS relapse, versus generalized mitochondrial oxidative stress is unclear. Future studies will seek to identify the mtDNA extrusion pathway to better understand this response in the setting of pediatric iNS.

## 4. Materials and Methods

**Sex as a biological variable:** All children (after consent or assent) presenting with iNS were enrolled consecutively and prospectively without consideration for sex. In the rat model, 6 animals (3 male, 3 female) were controls, and 6 animals (3 male, 3 female) were treated with PAN.

**Patient Cohort:** Children (both males and females) with nephrotic syndrome were prospectively and consecutively enrolled from the Children’s Hospital of Eastern Ontario (CHEO), Ottawa, Canada. The study was approved by the CHEO Research Institute Research Ethics Board (Protocol 18/168X) and is in accordance with the Declaration of Helsinki. Enrolment is ongoing, and for all patients enrolled, informed consent was obtained. Fifteen patients were initially enrolled, but one was excluded due to a missing urine sample. A further three patients were subsequently shown on follow up biopsy to have focal segmental glomerulosclerosis lesions and were thus excluded. This cohort contains 11 children with MCD on biopsy, or clinically suspected MCD in the absence of a biopsy. These children were heterogeneous in their presentation and response to steroid therapy. Following enrollment, clinical, biological, and anthropometric information was captured, and a urine sample was collected. Urine samples were subsequently collected from children again once they entered remission (defined as a urine protein to creatinine ratio of≤0.05 g/mmol).

**LEV Isolation:** LEVs were isolated using sequential centrifugation as described previously [15,38]. Briefly, urine or cell culture media was centrifuged at 2500× *g* for 10 min (to remove cellular debris and apoptotic bodies), the supernatant was then collected and centrifuged at 20,000× *g* for 20 min to pellet LEVs (while leaving exosomes in the supernatant). These LEVs were resuspended in sterile, pre-filtered (0.1 μm) phosphate-buffered saline (PBS) and frozen at –80 °C until used.

To isolate podocyte-derived LEVs from urine samples, a magnetic conjugation protocol was employed. Briefly, Anti-Podoplanin antibody (Human; Biolegend, San Diego, CA, USA, 337003) was conjugated to magnetic beads using the Magnetic Conjugation Kit (Abcam, Cambridge, UK, ab26989). LEVs were incubated with the Anti-Podoplanin antibody-magnetic conjugated beads for 4 h prior to being applied to a magnetic column. The Anti-Podoplanin antibody was diluted to 0.4 mg/mL with Mag Antibody diluent (Abcam), and 50 μL was added to a 20 μg vial of beads. The protocol was followed verbatim from the manufacturer.

**Nanoparticle Tracking Analysis (NTA):** NTA was performed as previously described [15]. Briefly, the ZetaView PMX110 Multiple Parameter Particle Tracking Analyzer (Particle Metrix, Meerbusch, Germany) was used. We used size-mode analysis with ZetaView software (version 8.02.28) following calibration with polystyrene beads (105 and 500 nm). Samples were analyzed at 11 camera positions with 2 s video length at 21 °C.

**mtDNA isolation from LEVs:** mtDNA levels in LEVs were measured as described previously, with modifications [24]. Briefly, isolated LEVs were treated with 2U of DNase-I to ensure coronal and attached DNA specimens were removed. Subsequently, LEVs were resuspended in 200 μL of PBS and treated with 20 μL of Proteinase K (Quiagen, Venlo, The Netherlands, 19131) and 2 μL of RNAse (10 mg/mL; Qiagen, Venlo, The Netherlands, 19101) and subjected to DNA isolation using a QIAmp DNA Mini Kit (Qiagen, Venlo, The Netherlands,) according to the manufacturer’s instructions. Total DNA specimens from the LEVs were then resuspended in Buffer AE and subjected to downstream quantitative polymerase chain reaction (qPCR) and ddPCR.

**mtDNA qPCR:** For qPCR analyses, samples were run in duplicate using mitochondrial-gene specific primers for the mitochondrial genes:
**Gene****Forward****Reverse****Human NADH Dehydrogenase Subunit 2 (ND2)**CCAAGGAATTCCCCTACACAGAAATTGCGAGAATGGTGGT**Human Cytochrome C Oxidase 2 (COX2)**AGACGCCACATCACCTATCACTTGGGCGTCTATTGTGCTT**Rat ND2**CCAAGGAATTCCCCTACACAGAAATTGCGAGAATGGTGGT

qPCR was performed using SYBR™ Green Master Mix (Applied Biosystems, Waltham, MA, USA, A25742) and 3 μL of DNA per reaction in a total reaction volume of 20 μL. Samples were run as follows: 2 min at 50 °C, 2 min at 95 °C followed by 40 cycles of 15 s at 95 °C, and 1 min at 60 °C, and the dissociation step (15 s at 95 °C, 30 s at 60 °C, and 15 s at 95 °C). The relative level of each mtDNA gene was calculated using the formula 2^−(Ct−X)^, that is a derivation of the 2^−ΔΔCt^ method. Results were normalized on the mean of the Ct for each primer.

For ddPCR reactions, a similar set-up was used as described above; however, with important differences. EvaGreen™ Master Mix (BioRad, Hercules, CA, USA, 1864033), a proprietary master mix specific to ddPCR reactions was used. Next, the DNA samples were added to the master mix preparations, including the same forward and reverse primers as highlighted above. Next, droplets were created using ddPCR Oil™ and the QX200 Droplet Generator™ (BioRad). Following droplet generation, the samples were moved to a PCR plate and run as follows: 5 min at 95 °C followed by 40 cycles of 30 s at 95 °C and 1 min at 60 °C. Thereafter, 4 °C for 5 min, 90 °C for 5 min, 4 °C for 10 min, and then 4 °C until removed from the machine. The PCR plate was read using the QX200 Droplet Reader™. This method allows for absolute quantification of copy number reported as copies/μL.

**RNA Isolation, cDNA creation, qPCR:** mRNA was isolated using an RNA-easy Mini Kit (Qiagen, Venlo, The Netherlands,, 74104) according to the manufacturer’s instructions. Following isolation, 200 ng mRNA was converted to cDNA using a high-capacity RNA to cDNA reverse transcription kit (ThermoFisher, Waltham, MA, USA, 4368814). The resultant cDNA was used for qPCR. qPCR was performed using SYBR™ Green Master Mix (Applied Biosystems, 4309155) and 2 μL of DNA per reaction in a total reaction volume of 20 μL. Samples were run as follows: 2 min at 50 °C, 2 min at 95 °C followed by 40 cycles of 15 s at 95 °C, 20 s at 58 °C, and 40 s at 72 °C, and the dissociation step (15 s at 95 °C, 30 s at 60 °C, and 15 s at 95 °C). The relative level of each mtDNA gene was calculated using the 2^−ΔΔCt^ method and normalized to glyceraldehyde 3-phosphate dehydrogenase (GAPDH) as a housekeeping gene.

**PRR and Effector Pathway qPCR:** Following cDNA creation, the following human genes were targeted: TLR9, cGAS, STING, IL-1ß, IL-6, and TNF-α. Their primer sequences are as follows:
**Gene****Forward****Reverse****TLR9**GCTGGACCTGAGTGAGAACTGAGTGAGCGGAAGAAGATGC**cGAS**TAACCCTGGCTTTGGAATCATAGCCGCCATGTTTCTTCTT**STING**ACTGTGGGGTGCCTGATAACCTTGCAGACTTTGTTTGCCA**IL-1ß:**ACGAATCTCCGACCACCACCTTGCAGACTTTGTTTGCCA**IL-6**GGTACATCCTCGACGGCATCTGTGCCTCTTTGCTGCTTTCAC**TNF-α**ATCTACCTGGGAGGCGTCTTGAGTGGCACAAGGAACTGGT

**Cell Culture:** Conditionally immortalized human podocytes (hPods) were obtained with permission of Prof. Moin Saleem (University of Bristol, Bristol, UK) [39]. Cells (5 × 10^4^) were grown in RPMI-1640 medium supplemented with 10% Fetal Bovine Serum (Gibco, Carlsbad, CA, USA, 12633012) and penicillin-streptomycin (1:100; Gibco, 15140122). Podocytes were proliferated at 33 °C in the presence of 10 U/mL Recombinant Human Interferon Gamma (γ-IFN; Invitrogen, RIFNG100). For induction of podocyte differentiation, cells were maintained at 38 °C for 13–14 days in the absence of γ-IFN with the experiment performed, in all cases, between days 13–14.

**Cell pre-treatment and toxin treatment:** Puromycin aminonucleoside (PAN; 25 μg/mL; Cayman Chemicals, Ann Arbour, MI, USA, 15509) is a well characterized and ubiquitously employed podocyte toxin and models iNS in the laboratory. Prednisolone pre-treatment occurred 1 h prior to treatment with PAN. Prednisolone (Pred, 1 μM; Cayman Chemicals, 20866) was resuspended in PBS, aliquoted, and used immediately or frozen. Dose selection was based on dose–response analysis and was consistent with prior studies. PAN treatments occurred for 1 h, or 24 h, as indicated. After treatment, the media was collected, and LEVs were isolated as described above. Cell plates were washed with ice cold PBS immediately after media collection and were frozen at −80 °C until protein isolation. A minimum of three biological replicates were employed for all conditions.

**Western blot Analysis:** Cell pellets were resuspended in 50 µL of radioimmunoprecipitation assay (RIPA) buffer, with 1:100 protease cocktail (PIC). Samples were mixed thoroughly, and the protein concentration was assessed using a BCA standard curve. Five micrograms of cell lysate were added to 6× Laemmli sample buffer and incubated at 95 °C (for Mitofusin-2 (MFN2), Dynamin-related protein 1 (DRP1), Translocase of the Outer Membrane 20 (TOM20), and β-actin probing) or 37 °C (for Oxidative Phosphorylation (OxPhos) protein probing). For each sample, protein was loaded onto a gel and subjected to sodium-dodecyl sulfate polyacrylamide gel electrophoresis (SDS-PAGE), with a stacking gel of 4% and a separating gel of 10% or 12%. This was followed by transfer to PVDF (OxPhos) or Nitrocellulose (MFN2, DRP1, TOM20). Blots were then washed thrice and blocked in 5% non-fat milk in tris-buffered saline with Tween-20 (TBS-T; pH 7.6). Thereafter, the blots were washed again, thoroughly, and then placed into primary antibody (diluted in 5% milk, as above) for overnight incubation at 4 °C. This was followed by incubation with 1:10,000 anti-rabbit IgG or anti-mouse IgG (Jackson ImmunoResearch, West Grove, PA, USA, 115-005-003). Chemiluminescence was induced by adding SuperSignal West Pico PLUS (ThermoFisher Scientific, 34580). Chemiluminescence was captured on a BioRad ChemiDoc Imaging system. Band densitometry was performed on ImageLab Software (V6.1).

Antibodies used were as follows:

**Mitofusin-2** (D2D10) Rabbit mAb #9482 1:1000

**TOM20** (D8T4N) Rabbit mAb #42406 1:1000

**DRP1** (D6C7) Rabbit mAb #8570 1:1000

**β-Actin** (13E5) Rabbit mAb #4970 1:3000

**OxPhos Antibody Cocktail** Mouse mAb #ab110411 1:5000

**Liquid chromatography and tandem mass spectrometry:** The MS/MS spectra were obtained using the following protocol [40]. Briefly, 50 μg of cell lysates in radioimmunoprecipitation assay buffer (150 mM NaCl, 1% Triton X-100, 0.5% sodium deoxycholate, 0.1% SDS, 50 mM Tris, pH 8.0) were isolated via single-pot protein precipitation (SP3) using 50% ethanol and proteolysis buffer (50 mM Ammonium Bicarbonate, 1:50 TrypLysC, 18 h, 37 °C) and analyzed on a Thermo Eclipse using Gas-phase Fractionation operating in Data-Independent Acquisition mode (GPF-DIA). Spectral libraries were searched in DIA-NN (v19.0) against the human proteome using a precursor mass tolerance of 20 ppm and fragment ion mass tolerance of 10 ppm. The search parameters allowed for up to 2 missed cleavages and a maximum of 2 post-translational modifications per peptide. Missing value imputation, transformations, and proteomic data visualizations were performed using in-house Python code (V3.13.3).

**Reactive Oxygen Species Kits (MDA and mitoROS):** MDA, a marker of oxidative stress, was analyzed according to manufacturer’s instructions (Cayman Chemicals, TBARS Assay Kit, 10009055). mitoROS was also assessed according to manufacturer’s instructions (Cayman Chemicals, Mitochondrial ROS Detection Kit, 701600).

TMRE assay: The TMRE mitochondrial membrane potential assay kit was purchased from Cayman Chemicals (701310). Briefly, cells were plated onto 96-well, black bottom plates and subjected to pre-treatment and toxin treatments as described. The assay was performed as described in the instruction booklet from the manufacturer.

**Seahorse Assay:** Oxygen consumption rates (OCR) were measured in hPods (1 × 10^4^ cells per well) using the Seahorse XF96 extracellular flux analyzer. Baseline OCR was recorded in the presence and absence of PAN, and thereafter Oligomycin (Oligo; Final Concentration 1 µM), Carbonyl cyanide-p-trifluoromethoxyphenylhydrazone (CCCP; Final Concentration 1 µM) and antimycin-A/rotenone (AA/R; Final Concentration 0.5 µM) were successively added to determine the impact on ATP-dependent (Oligo), maximal (CCCP), and non-mitochondrial (AA/R) respiration [41]. ATP Dependent respiration was calculated as Baseline OCR—Oligo OCR; Proton Leak was calculated as OCR Oligo—OCR AA/R; Basal OCR was calculated as Baseline OCR—AA/R; Maximum Respiration was calculated as CCCP OCR—Basal OCR; Non-mitochondrial OCR is equal to AA/R OCR. Results were normalized to cellular protein concentration (µg). Multiple, independent Seahorse runs were performed to collect the presented data (*n* = 3 for 1 h, *n* = 3 for 24 h).

**Experimental Design for Rat Studies:** Animal experiments were performed with adherence to, and approval by, the University of Ottawa Animal Care Committee (MEe-4256; Ottawa, ON, Canada). Experiments were conducted according to guidelines of the Canadian Council on Animal Care. Male and female Sprague-Dawley (SD) rats weighing 100–150 g were housed 2 animals per cage and had free access to food and water. Animals were randomly assigned to receive PAN or normal saline injection [37]. Of the 12 animals, 6 were injected with PAN (3 males, 3 females) and 6 were injected with saline (3 males, 3 females). The PAN injection was a single injection of 100 mg/kg and occurred on Day 0. Animals were housed thereafter for 9 days. Urine was obtained at Day 0 and 9. On Day 9, blood samples were obtained, and all rats were euthanized; kidneys were excised, decapsulated, and subsequently the renal cortex was dissected. Samples were taken for EM imaging. Albumin to creatinine ratio (ACR) was determined using the following kits from Abcam (Creatinine ab204537, Albumin ab235642).

**Transmission EM imaging:** All steps were performed at room temperature unless otherwise specified. After fixation, tissue portions were incubated for 1 h in a solution of 1.5% potassium ferrocyanide (Bioshop, Burlington, ON, Canada, PFC232.250) and 2% osmium tetroxide (prepared from 4% osmium tetroxide (aqueous) stock solution, Electron Microscopy Sciences, Hatfield, PA, USA, 19151). The tissue was then washed in ddH_2_O and incubated for 20 min in a 10 mg/mL solution of thiocarbohydrazide (Electron Microscopy Sciences, Hatfield, PA, USA, 21900). Following another wash in ddH_2_O, the tissue was incubated for 30 min in 2% osmium tetroxide. Finally, tissues were washed one last time in ddH_2_O before performing a series of dehydrations in increasing concentrations of ice-cold anhydrous ethanol (30%, 50%, 70%, 80%, 90%, and 3× 100%), where they were incubated in each concentration for 2 min. The tissue was then further dehydrated in propylene oxide (Sigma-Aldrich, St. Louis, MO, USA, 110205) for 5 min before being transferred to aluminum dishes containing Durcupan™ ACM resin (Electron Microscopy Sciences, Hatfield, PA, USA, 14040) overnight. The following day, the tissue was transferred to molds containing fresh Durcupan™ ACM resin and placed in a 55 °C oven for 72 h. Once removed from the oven, samples were sectioned with a diamond knife (80 nm) using an ultramicrotome. Sections were collected on 300-mesh copper grids (Electron Microscopy Sciences, Hatfield, PA, USA, G300-Cu) and imaged with a JEOL JEM1400-Flash electron microscope at 80 kV.

**Statistical Analysis:** Continuous variables were analyzed as mean (±standard deviation, SD) if normally distributed. When non-normally distributed, medians with interquartile ranges (IQR) were analyzed. *p*-values were generated using student *t*-tests or one-way analysis of variance (ANOVA) with multiple comparisons which were corrected using Tukey’s method if normally distributed. If non-normally distributed, data were analyzed for significance using Mann–Whitney U test or Kruskal–Wallis. Tests were performed using R (version 1.1.456) or Prism (version 9.4.1).

## Data Availability

This work does not contain algorithms, or big data sets. We, the authors, declare that the data supporting the findings of our study are available within this article. The authors will share details around animal models and experimental procedure with anyone who wishes to use these models. These models are highly published, with references herein. Please contact R.L.M., D.B., or C.R.J.K. for any further information.

## Supplementary Materials

The following supporting information can be downloaded at: https://www.mdpi.com/article/10.3390/ijms26157245/s1.
