# Supplementary material for: Extracellular Vesicle Mitochondrial DNA Reflects Podocyte Mitochondrial Stress and Is Associated with Relapse in Nephrotic Syndrome
Source: Int J Mol Sci. 2025 Jul 26;26(15):7245. doi: 10.3390/ijms26157245 (PMC12346890; doi:10.3390/ijms26157245)
Supplement: Supplementary file 1 [file ijms-26-07245-s001.zip › ijms-3752838-supplementary.pdf]

Supplementary Figure S1

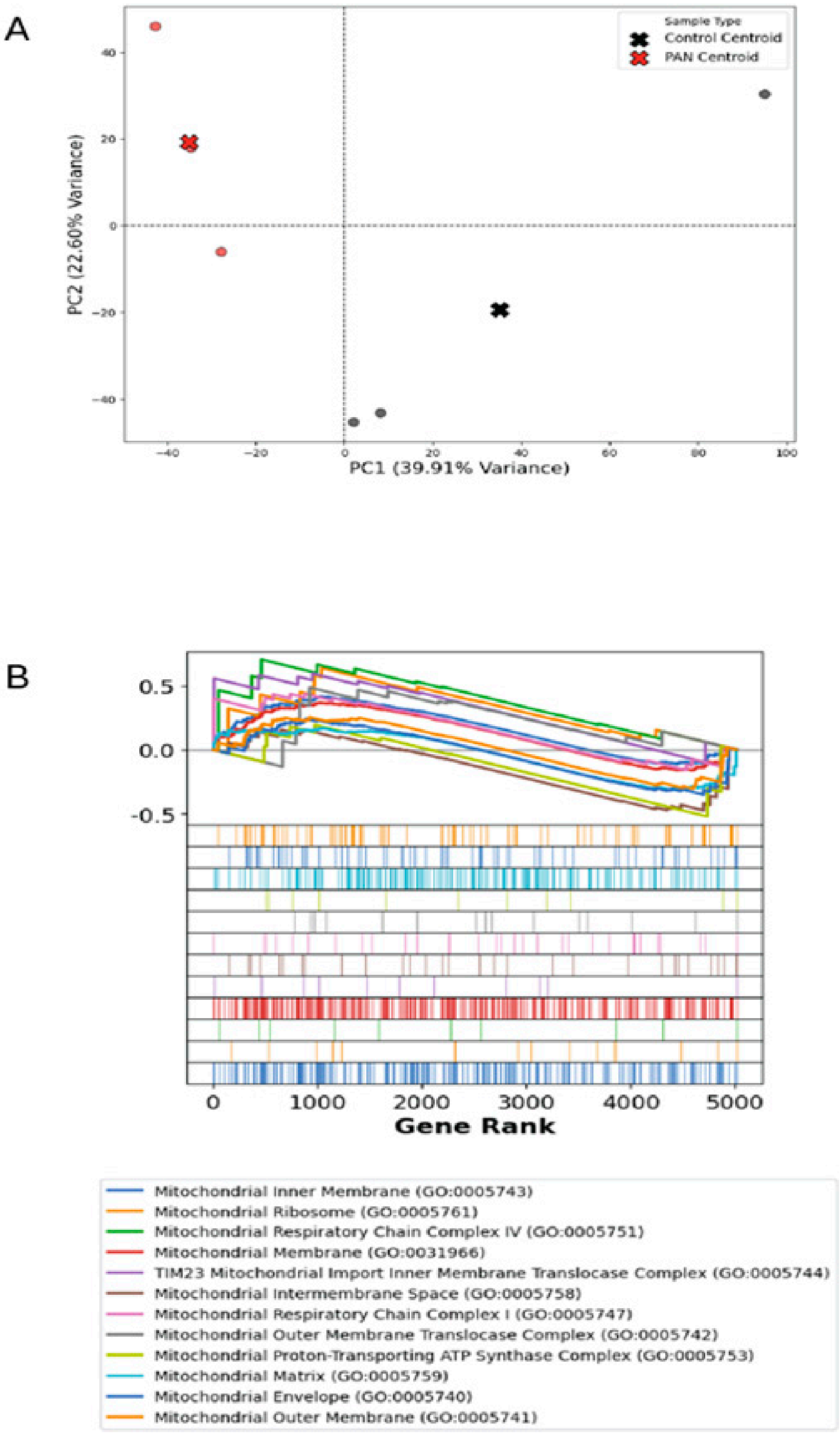

Figure S1. Proteomics of hPods revealed significant alterations in mitochondrial proteins. (**A**) Principal component analysis. (**B**) Gene set enrichment analysis.
